# Differences in Anticipated Interaction Drive Own Group Biases in Face Memory

**DOI:** 10.1371/journal.pone.0090668

**Published:** 2014-03-05

**Authors:** John Paul Wilson, Pirita E. See, Michael J. Bernstein, Kurt Hugenberg, Christopher Chartier

**Affiliations:** 1 Department of Psychology, University of Toronto, Toronto, Ontario, Canada; 2 Department of Psychology, Miami University, Oxford, Ohio, United States of America; 3 Department of Psychological and Social Sciences, Pennsylvania State University Abington, Abington, Pennsylvania, United States of America; 4 Department of Psychology, Ashland University, Ashland, Ohio, United States of America; University Medical Center Goettingen, Germany

## Abstract

According to much research, the Own Group Bias (OGB) in face memory occurs as a consequence of social categorization – ingroup members are more likely than outgroup members to be encoded as individuals and remembered well. The current work is an examination of the role of anticipated future interaction in the OGB. We conducted two studies showing that anticipated interaction influences group-based face memory. In Study 1, we provided correlational evidence that beliefs about the amount and importance of future interaction one will have with racial outgroup members is associated with the OGB, such that people expecting more interaction with outgroup members show a reduced OGB. In Study 2, we manipulated expectations about future interactions with lab-created groups and observed that high levels of anticipated future interaction with the outgroup eliminated the OGB. Thus, social group categorization drives face memory biases to the extent that group membership affords the expectation of interpersonal interaction.

## Introduction

The Own Group Bias (OGB), or the tendency to better recognize faces of ingroup members than of outgroup members, has been of longstanding interest to psychologists [1], [2]. Although this OGB in face memory was originally studied in the context of race (see [3] for a review), extensive research has demonstrated that the OGB occurs across a variety of ingroup-outgroup dimensions, such as age [4], sex [5], sexual orientation [6], and even minimal groups [7]. Further, categories of choice such as university affiliation [8] and even completely arbitrary categories, such as experimentally-created teams [9] or personality types [7] can generate OGBs.

Recent evidence indicates that Own Group Biases occur across so many categories largely because categories can signal the differential need to individuate faces [10]. This *group as signal* hypothesis posits that rather than group membership *per se,* it is the information conveyed regarding what the group affords the perceiver that underlies individuation [11]. Ingroups support us and fulfill our needs [12], which means that attending to and encoding information about ingroup members can be functional across a variety of contexts.

### Individuation, Processing Goals, and Anticipated Future Interaction

The tendency to individuate ingroup faces has clear analogues in the social psychological literature on person perception. Although the specific cognitive mechanisms by which faces versus semantic information about others are encoded certainly differ [13], the motivation to attend to unique information about others, and especially outgroup members, appears central to person perception across a variety of contexts. Whereas categorizing others is a highly efficient cognitive process, attending to and processing individuating information tends to be cognitively costly, and thus typically requires both motivation and cognitive capacity [14]–[16].

Although outgroup members are commonly treated categorically by default, outgroup members are individuated when one is *outcome dependent* upon them. For example, making perceivers outcome dependent on a member of a stigmatized outgroup can enhance individuation, even in situations that commonly elicit categorical treatment. Neuberg and Fiske [17] found that people became highly likely to individuate a formerly hospitalized schizophrenic person when they believed that cooperating with this individual could earn them a cash prize. More importantly for the current work, anticipating a future interaction with outgroup members can also trigger individuation. For example, Devine, Sedikides, and Fuhrman [18] found that people engaged in more individuation (i.e., remembered more information; processed the target more deeply) of targets with whom they expected an interaction than for targets associated with other processing goals (e.g., self-comparison, memory). Consistent with these findings that anticipated interaction with others appears to facilitate individuation, expecting to interact with others over time facilitates positive interactions in a number of contexts, such as in economic games [19], business transactions [20], and even experimentally determined dating relationships [21].

Extending this logic, we believe that perceivers’ goals regarding others are important in explaining Own Group Biases. In particular, one typically stable component of OGBs is that people will commonly expect more frequent (and more important) interactions with ingroup members than with outgroup members. Indeed, ingroups are commonly seen as stable sources of physical and psychological support, making them both salient and subjectively important [12]. Given that people expect both more frequent and more valued interactions with ingroup members, we believe that this baseline difference in anticipated interactions, and therefore outcome dependency, may be one reason why Own Group Biases exist across a variety of dimensions, even when holding perceiver expertise constant.

Some existing evidence provides indirect support for this hypothesis regarding anticipated future interactions playing a causal role in the Own Group Bias. Consider recent research from Van Bavel and Cunningham [22] who found an OGB when participants were assigned to teams (e.g., ‘Suns’ versus ‘Moons’). However, the authors also indirectly manipulated whether participants believed that they would interact more with ingroup members than outgroup members. When participants were instructed they were ‘soldiers’ who would ‘serve the needs of’ the ingroup, presumably creating an expectation of frequent and valued interaction with the ingroup, the typical OGB was observed. However, when participants were instructed that they were ‘spies’ who would ‘infiltrate’ the outgroup while ‘remain[ing] loyal to the’ ingroup, presumably creating an expectation of frequently and valued interaction with both ingroup and outgroup members, the OGB was eliminated via an increase in outgroup recognition. Although this study was not designed to directly test whether expectations about future interactions play a causal role in creating and eliminating OGBs, the results are certainly congruent with this hypothesis.

This work serves as indirect evidence that anticipated, valuable interactions may play a causal role in Own Group Biases. Not only do people generally expect more interactions with ingroups than outgroups, but manipulations that affect expectations about future interactions also influence the OGB. The current work, however, is the first designed to directly test the hypothesis that OBGs occur, in part, due to expectations of more frequent and subjectively important interactions with the ingroup, relative to the outgroup.

### The Current Research

In two studies, we tested the hypothesis that differences in anticipated future interactions with ingroup and outgroup members play a causal role in the Own Group Bias. In the first study we focused on the Own Race Bias, a commonly researched OGB for which there is a strong existing basis for expectations of differential interactions (i.e., *de facto* racial segregation). In this correlational study, we measured individual differences in expectations of greater frequency and importance of own-race (versus cross-race) interactions, and provided novel evidence that these individual differences in anticipated interactions predict the magnitude of the Own Race Bias across participants. In Study 2, we employed an experimental design in which we created an Own Group Bias in the laboratory by assigning participants to bogus ‘personality types’ in the laboratory. By creating groups in the laboratory [7], [9], we were able to directly manipulate whether participants expected more interactions with the ingroup than with the outgroup, or equivalent interactions with ingroup and outgroup members. As predicted, we found that whereas the OGB is observed when perceivers expect more ingroup than outgroup interactions, it is eliminated when perceivers expect equivalent interactions with the ingroup and the outgroup.

## Study 1

We conducted Study 1 to provide initial, correlational support for the anticipated interaction hypothesis. Although our hypothesis is a causal one, we thought it important to initially show that the magnitude of the Own Race Bias is predicted by perceivers’ expectations about own- versus cross-race interactions. This is because the Own Race Bias is by far the most commonly researched OGB in the literature [3], [10], [11], and most existing theory regarding OGBs has been designed to address this Own Race bias (see [10] for a review). Thus, it was first important to demonstrate that the Own Race Bias could be predicted by perceivers’ expectations about future own- versus cross-race interactions.

To address this question, in Study 1 we asked White participants to first complete a recognition task for White and Black faces, adapted closely from commonly employed paradigms used to study the Own Group Bias. This was followed by a brief measure assessing the extent to which participants believe they will have important future interactions with both White (own-race) and Black (cross-race) people. We predicted that the Own Race Bias would be strongest among participants who expected to have more future interactions with Whites than Blacks.

### Method

#### Ethics Statement

This study was approved by the Institutional Review Board at Pennsylvania State University, Abington Campus. All participants provided written informed consent prior to participation in the study.

#### Participants and design

A convenience sample of forty-six White undergraduates (24 female) participated for partial course credit. Two participants exhibited below chance recognition scores and were excluded from analyses. Participant gender did not moderate the results and will not be discussed further (though some research has found own-group effects in memory based on participant gender, this particular group distinction has proven quite inconsistent in producing such effects [3], and we did not have *a priori* expectations for differential effects based on gender.

Target race was manipulated within-participants, and the predicted anticipated interaction with Whites and Blacks was measured as an individual difference. The dependent variable was recognition sensitivity (*d′*), which is a standard signal detection measure of recognition that incorporates both hits and false alarms.

### Materials and procedure

Participants arrived at the laboratory for a study on face processing. After providing informed consent, participants were seated in separate computer cubicles, and completed the face recognition task, which consisted of two phases. They then completed the anticipated interaction questionnaire. Finally, participants were debriefed and thanked.

#### Face encoding phase

First, participants viewed 40 neutral expression male faces (20 White; 20 Black) one at a time, on a computer screen. Face stimuli were harvested from various publicly available online sources. They were cropped at the neck, eliminating jewelry or distinctive hairstyles. Stimuli were presented in grayscale, sized to approximately 5.7×3.8 cm, and were presented on white backgrounds. Faces were displayed in random order for 3s each, with an inter-stimulus interval of 500 ms, during which a fixation point appeared onscreen.

#### Face recognition phase

After a 5-minute distraction task intended to clear working memory, participants viewed 80 male faces (40 White; 40 Black) in random order. Half of these faces were viewed in the encoding phase, and half were new (i.e., not seen during the encoding phase). Participants’ task was to indicate via keystroke whether they had seen each face before or not. Each face remained onscreen until the participant rendered a response.

#### Anticipated interaction value items

Finally, participants answered questions regarding their expected interactions with Whites and Blacks. There were two primary items, each of which was asked separately in reference to Whites and Blacks, for a total of four questions. Participants rated the extent to which “it is important for me to recognize the faces of White (Black) people,” on a Likert scale ranging from 1 (not at all important) to 5 (very important), designed to measure the differential perceived importance of ingroup versus outgroup individuation. For the other item, participants were asked to estimate “in the next week, what percentage of your interactions do you expect to be with White (Black) people,” which was designed to measure the differential expectations about the frequency of interactions with the ingroup versus the outgroup.

### Results and Discussion

Of initial interest was whether participants demonstrated an Own Race Bias, observed as better own-race than cross-race recognition. To investigate this, we first calculated recognition sensitivity (*d′*) scores for own-race and cross-race faces, separately for each participant. As expected, our participants exhibited the ORB, as own-race faces (*M* = .94, *SD* = .53) were recognized more accurately than other-race faces (*M* = .70, *SD* = .49), *t*(43)  = 2.45, *p* = .02, *d* = .47.

Of primary interest was whether the Own Race Bias was associated with levels of anticipated interaction with own- and cross-race targets. To address this question, we computed a composite measure of anticipated interaction questions. We first computed White-Black difference scores on both anticipated interaction value items, such that lower scores indicated a greater tendency to expect a high frequency of interaction with Blacks relative to Whites as well as viewing interactions with Black as subjectively important relative to Whites. We then standardized scores for each of the items. The two measures were marginally correlated with one another [*r*(43) = .26, *p* = .088)]. Next, we computed a measure of ORB (White minus Black recognition) for each participant, such that higher scores indicate more accurate recognition for own-race than other-race faces.

As predicted, participants reported a strong tendency to see interactions with (ingroup) Whites as more important than with (outgroup) Blacks, *t*(43)  = 3.15, *p*<.01, and to expect a higher percentage of their interactions in the next week to be with Whites (*M* = 73.05, *SD* = 19.51) than Blacks (*M* = 19.08, *SD* = 15.91), *t*(43)  = 13.42, *p*<.01. Finally, we regressed the Own Race Bias score on perceived importance of remembering and anticipated interaction scores in a multiple regression. This model did account for a significant amount of variance in the ORB, *F*(2,41)  = 3.33, *p* = .046. However, examining the predictors separately, anticipated interaction was uniquely associated with a reduction in the ORB, *β* = .361, *t*(41)  = 2.40, *p* = .021. Perceived importance of remembering same- vs. other-race faces did not predict the ORB, *β* = .042, *t*(41)  = .28, *p* = .78. In other words, as White participants report believing that they are increasingly likely to have future interactions with Black people, the ORB is diminished.

To our knowledge, this is the first study to directly test the extent to which expectations about future interactions can predict the magnitude of the Own Race Bias. Despite this supportive preliminary evidence for a role of anticipated interactions in the ORB, it is possible that the relationship is the product of a third variable. Indeed, expectations about cross-race interaction may themselves be a product of past experience with cross-race individuals. A large body of prior research has argued that the ORB can be caused by differential contact with own-race and cross-race targets [23]. As such, it could be that differences in prior interracial contact cause both the ORB and the predictions of future intra- versus inter-racial contact. However, if results analogous to those of Study 1 emerged for lab-created groups in response to a manipulation of anticipated interaction, differential past experience with racial groups cannot account for the effect. The second study was designed to rule out this alternative hypothesis. Further, because it was only anticipated interaction and not perceived importance that predicted the ORB, we decided to focus primarily on anticipated interaction as our dependent measure in Study 2.

## Study 2

In Study 2, we explicitly manipulated anticipated intergroup contact. If differential expectations about interactions with ingroup and outgroup members plays a causal role in the Own Group Bias, we should be able to demonstrate this directly by showing that a manipulation of anticipated intergroup interactions affects the OGB.

Before testing this hypothesis, we conducted a pilot study designed to establish that participants expect more high-quality interaction with ingroups than with outgroups, even when those ingroup/outgroup distinctions are experimentally created. To test this hypothesis, we relied on a technique adapted from previous research on the OGB [7], [24], using a bogus personality test to assign participants to one of two personality groups. After pilot study participants were assigned to groups, we asked participants to predict how many ‘of the next ten people they interact with’ would be ingroup members (i.e., fellow Red [Green] personality types), and how many would be outgroup members (i.e., personality type outgroup), and whether ingroup or outgroup members would be more important to ‘future life experiences.’ As predicted, participants reliably believed that they would interact more with ingroup than with outgroup members, and that ingroup members would be more important to future life experiences, *p*s <.01. Thus, it appears that even for experimentally created groups, people expect more frequent and more important interactions with ingroup members.

Based on these findings, it seemed sensible to proceed using an experimentally created group procedure to test whether the OGB occurs in part due to differential expectations about ingroup and outgroup interactions. In Study 2, White participants received bogus feedback about their personality type (again, assigning them to ‘Red’ or to ‘Green’ personality types). Once participants were assigned to a bogus personality group, we also used additional task instructions to manipulate beliefs about future interactions with ingroup versus outgroup members. Participants assigned to the Ingroup Frequent condition were led to believe that they would have more frequent interactions with ingroup than with outgroup members. We predicted that this Ingroup Frequent condition would mirror participants’ default expectations of more and greater quality contact with ingroups (see the pilot data above), eliciting an Own Group Bias. Participants assigned to the Equal Frequency condition, however, were led to believe that they would have equally frequent interactions with ingroup and outgroup members. We predicted that the OGB would be attenuated in this Equal Frequency condition relative to the Ingroup Frequent condition – there should be no deficit in individuation for outgroup members if one expects frequent, meaningful interactions with them.

### Method

#### Ethics Statement

This study was approved by the Institutional Review Board at Miami University. All participants provided written informed consent prior to participation in the study.

#### Participants and design

A convenience sample of fifty-four White undergraduates (41 Women) participated for course credit. As is often the case with samples at this university, the gender distribution was unbalanced. Consistent with past research [7], gender did not moderate the results and will not be discussed further. This study employed a 2(Outgroup interaction: infrequent vs. equal) × 2(Ingroup: red, green) × 2(Target group membership: red, green) mixed design, with target group membership manipulated within-subjects. The primary dependent variable was recognition sensitivity (*d′*).

### Materials

Materials consisted of 80 pictures of White college-age male faces, all facing forward and displaying neutral facial expressions. As in Study 1, these face stimuli were harvested from various publicly available online sources. They were cropped at the neck, eliminating jewelry and distinctive hairstyles. The stimuli were presented in grayscale, sized to approximately 5.7 × 3.8 cm, and presented on 7.6×7.6 cm red and green backgrounds. The color name (‘Red’ or ‘Green’) was inscribed in white letters at the bottom of the red and green backgrounds, respectively.

### Procedure

Participants were seated in private computer cubicles and instructed that they would engage in a study on interpreting social stimuli. The experiment consisted of three phases, all of which were administered via the computer.

#### Personality assessment and anticipated interaction manipulation

First, participants were assigned to Red or Green personality groups in a procedure adapted from Bernstein and colleagues ([7]; see also [24]). The personality assessment was followed by the manipulation of anticipated future interaction. Participants in the Ingroup Frequent condition were informed that the majority of their future interactions would be with people sharing a personality group with them, whereas outgroup interactions would occur more rarely. Participants in the Equal Frequency condition, on the other hand, were told that, though group membership was meaningful, they would generally have equal interactions with both groups (see Appendix S1).

#### Learning phase

Next, participants completed the learning phase, which was nearly identical to that used in Study 1, but with 40 White faces on Red or Green backgrounds. Whether each face appeared as a target during encoding or as a distracter during recognition, and whether each face appeared on a red or a green background was counterbalanced across participants.

#### Face recognition phase

After a 5-minute distraction task, participants completed the recognition phase. Participants viewed 80 faces (40 Red group; 40 Green group), half of which they had seen during the learning phase, and indicated for each face whether they had seen it before or not.

### Results and Discussion

Here, we tested whether we could conceptually replicate the results of Study 1, but in a situation where we both randomly assigned participants to arbitrary ingroups and manipulated beliefs about future intergroup contact. We hypothesized that an OGB would emerge when participants believed they would interact more frequently with ingroup than outgroup members. Of primary interest was whether the OGB would be attenuated when the frequency of ingroup and outgroup contact was believed to be equivalent.

**Figure 1:**
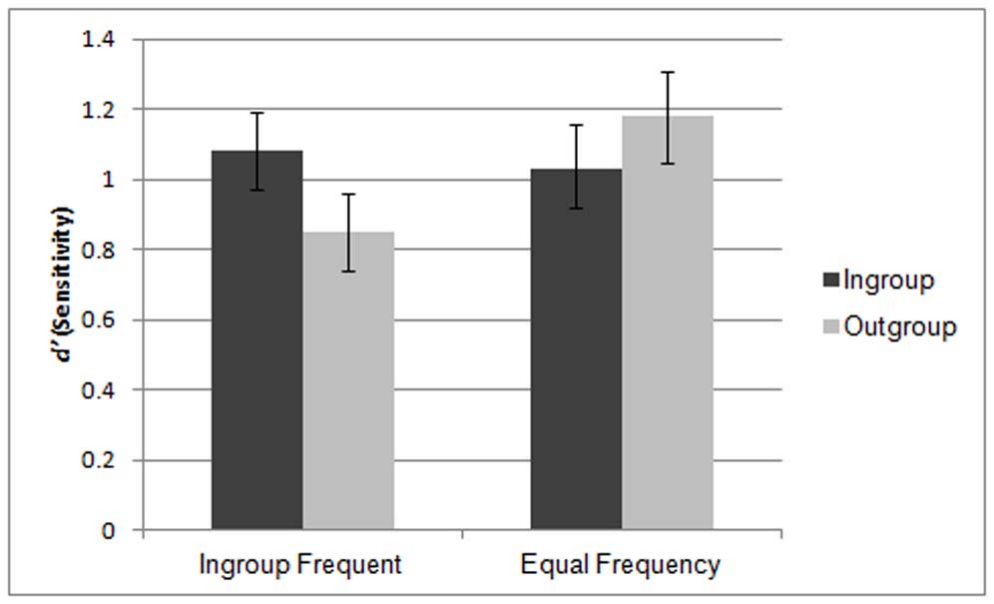
Ingroup and outgroup recognition as a function of anticipated interaction feedback.

*D'* scores were subjected to a 2(Anticipated interaction: Ingroup Frequent, Equal Frequency) × 2(Target group membership: ingroup, outgroup) mixed ANOVA, with repeated measures on the second factor. As predicted, we observed a significant interaction between target group membership and anticipated interaction, *F*(1, 52)  = 4.46, *p* = .04, η_p_^2^ = .08 (see Figure 1). Variance between groups was homogenous, *p*>.6.

We decomposed this interaction by conducting planned comparisons of estimated marginal means. In the Ingroup Frequent condition, participants showed marginally better recognition for ingroup targets (*M* = 1.08, *SE* = .11) than for outgroup targets (*M* = .85, *SE* = .12), *p* = .07. However, in the Equal Frequency condition, the OGB was eliminated, as outgroup recognition (*M* = 1.18, *SE* = .13) was descriptively albeit non-significantly better than ingroup recognition (*M* = 1.03, *SE* = .11), *p* = .*25*. Comparing across conditions, the group interaction manipulation had no effect on ingroup recognition, *p* = .78. However, believing that outgroup interaction was equivalent to ingroup interaction marginally improved outgroup recognition over the low-frequency condition, *p* = .08.

As predicted, beliefs about the frequency of intergroup interaction influenced the OGB: a significant interaction of target group membership and anticipated interaction emerged. Participants who were led to expect more interactions with the ingroup than the outgroup showed a marginally significant OGB. However, participants who believed that they were no more likely to interact with ingroup than outgroup members did not show the OGB, and this occurred largely due to an increase in outgroup recognition. Though this increase in outgroup recognition did not reach conventional significance, it is quite clear that participants in the Equal Interaction condition did not show the typical OGB, and in fact showed descriptively better recognition for outgroup than ingroup faces. Nevertheless, given the marginal nature of the planned comparisons interpretations of the data below the level of the significant interaction should be done with some caution.

## General Discussion

In two studies, we demonstrated that expectations about intergroup interactions may play an important role in the Own Group Bias. Participants who expect more high-quality interactions with the ingroup than the outgroup – measured in Study 1, manipulated in Study 2 – showed a greater OGB than did participants who expect more equal interactions between these groups. We found these effects both as a function of individual differences based on stable beliefs about race, as well as through an experimental manipulation using novel groups. This research shows that beliefs about the importance of a group to one’s future interactions may be an important determinant of face memory.

This work suggests a novel method for improving outgroup recognition as well. Previous work has shown that instructing participants to focus on the individuating qualities of other-race faces can reduce the OGB [25], [26]. The current work shows that outgroup recognition may also be improved by changing beliefs about future interactions. Critically, it is clear that participants, by default, expect more high-quality interactions with ingroup members than with outgroup members. In Study 1, participants showed a strong average tendency to expect more interactions with racial ingroup members than racial outgroup members, and based on our pilot data for Study 2, participants expected more frequent and more important contact with even experimentally generated ingroup members. However, the results of Study 2 demonstrate that these beliefs are malleable, and under the right circumstances, changing these beliefs about intergroup contact can affect face recognition.

The present work builds on a social cognitive tradition focusing on factors influencing social memory. Fiske and Taylor [27], for instance, proposed a depth of processing continuum – from simple recall instructions to impression formation goals to anticipated interaction. Analogous anticipated interaction effects have been demonstrated by numerous researchers. Srull and Brand [28], for example, found that anticipated interaction attenuated list-length effects on memory for behaviors. Erber and Fiske [29] found that outcome dependency with a fellow participant led to enhanced attention to inconsistent information. Anticipated interaction also increases the number of attributions made for behaviors in social interactions [30]. Finally, data recently reported by Baldwin and colleagues [31] showed that participants expecting to rely on an other-race person in a subsequent interaction showed greater other-race recognition before their anticipated interaction task. Though some work has shown that own-race biases in social memory are difficult to moderate by anticipated interaction [32], our research shows that people do better individuate outgroup members if they anticipate interactions with members of the outgroup.

All of this research has functioned to establish that processing goals in a specific context influence social memory. Distinct from this past work, the present data show that ongoing assumptions about future interactions with an outgroup as a whole can influence basic processes (such as recognition) in face perception. As we have clearly shown, individuals show a default expectation that they will interact more with ingroup members than outgroup members. However, given an expectation that their future interactions will be more mixed, outgroup recognition is improved. This is important because it suggests that beliefs about relations between social groups will drive face memory in and outside of the lab. Because of the generality of our measures and manipulations, we believe that this work is an important and novel contribution to OGB research.

A growing corpus of research has shown that the OGB is driven largely by social motivational affordances of ingroups relative to outgroups [32], [33], [34]. The current work extends our understanding of this phenomenon by establishing a role of differences in anticipated future interactions. Study 2 was particularly important in demonstrating that the mechanism of this effect is distinct from mere differences in prior contact with ingroup and outgroup members and the resulting expertise differences, as expertise was held constant. Taken together, these studies represent a novel addition to the literature showing that group membership is a signal that transmits critical information regarding the need to individuate another person’s face. When group membership becomes non-diagnostic of future interactions, however, it may no longer exert an influence over face memory. As such, it is what the group affords rather than group membership *per se* that guides efforts to individuate faces.

## Supporting Information

Appendix S1 **Study 2 Anticipated Interaction Instructions Manipulation.**
(DOCX)
