# Supplementary material for: Differences in Anticipated Interaction Drive Own Group Biases in Face Memory
Source: PLoS One. 2014 Mar 5;9(3):e90668. doi: 10.1371/journal.pone.0090668 (PMC3944439; doi:10.1371/journal.pone.0090668)
Supplement: Appendix S1 — Study 2 Anticipated Interaction Instructions Manipulation. (DOCX) [file pone.0090668.s001.docx]

**Appendix S1**

Ingroup Frequent

These personality assignments are important for understanding the kinds of people with whom we choose to enter into relationships. This test predicts the kinds of people we are going to be friends with, work with, and the people with whom we choose to spend our lives with. In this case, people who responded to the test as you did tend to interact mostly with personality ingroup members rather than outgroup members. This means that RED people tend to spend time with RED people while GREEN people tend to spend most of their time with GREEN people. Though this may be different for some people, you will have very few intermixed relationships and should expect to interact primarily with ingroup personality types with the most regularity.

Equal Frequency

These personality assignments are important for understanding the kinds of people with whom we choose to enter into relationships. This test predicts the kinds of people we are going to be friends with, work with, and the people with whom we choose to spend our lives with. In this case, people who responded to the test as you did tend to interact equally with personality ingroup members and outgroup members. This means that RED people tend to spend time with RED people and GREEN people in equal amounts, and the same is true for GREEN people spending equal times with RED and GREEN people. Though this may be different for some people, you will have many intermixed relationships and should expect to interact with both personality types with equal regularity.
